# Noncalcemic 20-hydroxyvitamin D3 inhibits human melanoma growth in *in vitro* and *in vivo* models

**DOI:** 10.18632/oncotarget.14193

**Published:** 2016-12-26

**Authors:** Cezary Skobowiat, Allen S.W. Oak, Tae-Kang Kim, Chuan He Yang, Lawrence M. Pfeffer, Robert C. Tuckey, Andrzej T. Slominski

**Affiliations:** ^1^ Department of Dermatology, University of Alabama at Birmingham, AL, USA; ^2^ Department of Pharmacodynamics and Molecular Pharmacology, Faculty of Pharmacy, Collegium Medicum, Nicolaus Copernicus University in Torun, Poland; ^3^ Department of Pathology and Laboratory Medicine, and the Center for Cancer Research, University of Tennessee Health Science Center, Memphis, TN, USA; ^4^ School of Chemistry and Biochemistry, The University of Western Australia, Crawley, WA, Australia; ^5^ Laboratory Service of the VA Medical Center, Birmingham, AL, USA; ^6^ Comprehensive Cancer Center Cancer Chemoprevention Program, University of Alabama at Birmingham, AL, USA; ^7^ Nutrition Obesity Research Center, University of Alabama at Birmingham, AL, USA

**Keywords:** melanoma, pre-clinical, SKMel-188, vitamin D, mice

## Abstract

A novel pathway of vitamin D_3_ (D_3_) metabolism, initiated by C20-hydroxylation of D_3_ by CYP11A1, has been confirmed to operate *in vivo*. Its major product, 20(OH)D_3_, exhibits antiproliferative activity *in vitro* comparable to that of 1,25(OH)_2_D_3_, but is noncalcemic in mice and rats. To further characterize the antimelanoma activity of 20(OH)D_3_, we tested its effect on colony formation of human melanoma cells in monolayer culture and anchorage-independent growth in soft agar. The migratory capabilities of the cells and cell-cell and cell-extracellular matrix interactions were also evaluated using transwell cell migration and spheroid toxicity assays. To assess the antimelanoma activity of 20(OH)D_3_ *in vivo*, age-matched immunocompromised mice were subcutaneously implanted with luciferase-labelled SKMel-188 cells and were randomly assigned to be treated with either 20(OH)D_3_ or vehicle (*n*=10 per group). Tumor size was measured with caliper and live bioimaging methods, and overall health condition expressed as a total body score scale. The following results were observed: (i) 20(OH)D_3_ inhibited colony formation both in monolayer and soft agar conditions, (ii) 20(OH)D_3_ inhibited melanoma cells in both transwell migration and spheroid toxicity assays, and (iii) 20(OH)D_3_ inhibited melanoma tumor growth in immunocompromised mice without visible signs of toxicity. However, although the survival rate was 90% in both groups, the total body score was higher in the treatment group compared to control group (2.8 vs. 2.55). In conclusion, 20(OH)D_3_, an endogenously produced secosteroid, is an excellent candidate for further preclinical testing as an antimelanoma agent.

## INTRODUCTION

Melanoma, the deadliest type of skin cancer, is responsible for 75% of skin cancer-related deaths with incidence continually rising [1]. In 2015, the estimated prevalence and mortality of cutaneous melanoma in the USA were 73,870 and 9,940, respectively [2]. Melanoma is driven by the combination of genetic and environmental factors. Ultraviolet (UV)-induced DNA damage [3–5], loss-of-function mutations in *CDKN2A*, and inactivating variants of melanocortin 1 receptor gene (MC1R) that are associated with poor tanning ability [6], as well as red hair all play roles in increased risk of melanoma occurrence [7]. In addition, evidence is accumulating that defects in vitamin D signaling can affect melanomagenesis, tumor progression, and final disease outcome [8–15].

Current treatment options for advanced cutaneous melanoma include surgical metastasectomy [16], radiation therapy [17], immunotherapy, and targeted therapy against the mitogen-activated protein kinase (MAPK) and *c-KIT* pathways [18–24]. Although many of these targeted therapeutic modalities are beneficial, they tend to be costly and are associated with many adverse effects. In addition, molecularly targeted therapies require mutations in targeted genes such as *BRAF* or *c-KIT* which may not be present in subsets of melanoma patients, rendering these treatments ineffective. Finally, disease recurrence often occurs due to development of resistance (discussed in [24–26]).

Vitamin D_3_ undergoes a two-step activation via hydroxylation in the liver at C25 and in the kidney at C1α, resulting in the production of its active form, 1,25(OH)_2_D_3_ (calcitriol) [27, 28]. The same sequence of D_3_ activation occurs in the skin [29, 30]. 1,25(OH)_2_D_3_ is not only implicated in regulating calcium levels, but also is involved in promoting the expression of genes that are anti-inflammatory, anti-proliferative, anti-angiogenic and anti-carcinogenic [27–29, 31–34]. The anticancer properties of D_3_ [14, 33, 35, 36] served as the rationale for an ongoing phase II clinical trial in Australia evaluating the use of vitamin D as an adjuvant therapy for treating melanoma with a high risk of recurrence [37].

Having vitamin D levels at the higher end of the physiological range may be beneficial. However, anticancer activities of vitamin D, particularly those of 1,25(OH)_2_D_3_, appear to require pharmacological doses that cause hypercalcemia. At a dose as low as 0.1 μg/kg for 1,25(OH)_2_D_3_, calcium depositions cause damage to the vital organs [38]. Thus, the hypercalcemic effects of 1,25(OH)_2_D_3_ at therapeutic doses severely limit its utility as an anti-cancer agent.

Mammalian CYP11A1, (also known as cytochrome P450scc), encoded by the *CYP11A1* gene, can hydroxylate vitamin D_3_ at positions C17, C20, C22 and C23 to produce several D_3_ hydroxyderivatives with 20(OH)D_3_ being a major product of the pathway [39–43]. Many of these novel hydroxylated vitamin D_3_ derivatives have been found in various organs, including the skin [44–46]. These hydroxyderivatives display anti-tumorigenic and anti-melanoma activities comparable to those of 1,25(OH)_2_D_3_ [47–52], and are noncalcemic in rodents at therapeutic doses as high as 60 μg/kg [50, 53, 54].

The main goal of this project was to test the hypothesis that 20(OH)D_3_ has anticancer activity equivalent to 1,25(OH)_2_D_3_on the growth, proliferation and progression of melanoma cells *in vitro*, and displays such properties at therapeutic doses that are noncalcemic using an *in vivo* model with immunocompromised mice.

## RESULTS

### Antimelanoma activity of 20(OH)D_3_ with *in vitro* models

**Figure 1:**
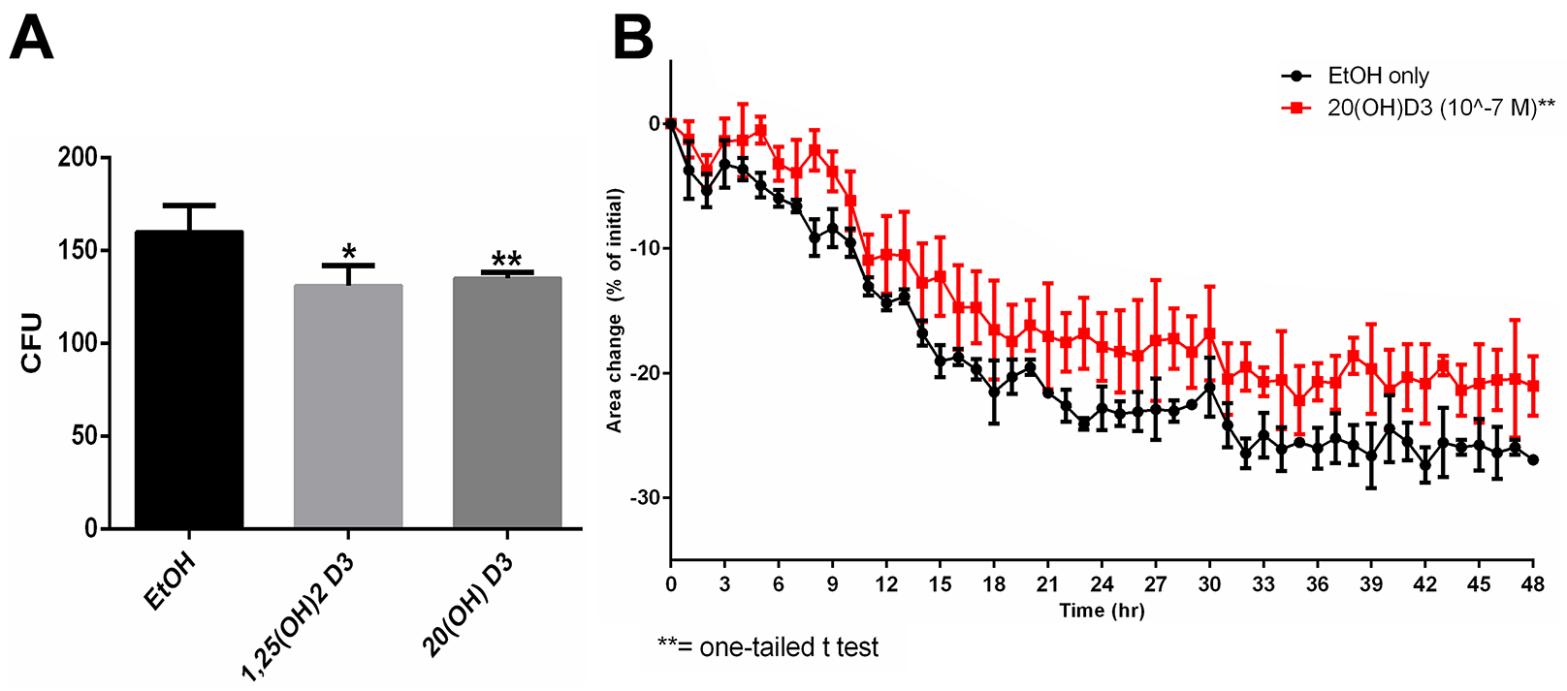
Effects of active forms on vitamin D on colony formation and spheroid toxicity assay in melanoma cells **A**. SKMel-188 cells were seeded at 14 cells/cm^2^ and incubated with 10^-7^ M 20(OH)D_3_ or 1,25(OH)_2_D_3_(or ethanol vehicle) for 14 days. Colonies > 0.2 mm were counted. **B**. Assessment 20(OH)D_3_'s cytotoxicity using the spheroid toxicity assay where area changes are indicative of migratory capability, cell-cell interactions and cell-ECM interactions. A smaller area change is indicative of greater cytotoxicity. SKMel-188 cells (50,000 cells/well) were printed into spheroids. Subsequently, they were incubated in the presence of 1,25(OH)_2_D_3_ or 20(OH)D_3_ (or ethanol vehicle as a control) at concentrations of 10^-7^ M (only 20(OH)D_3_ is shown here, since 1,25(OH)_2_D_3_ had no significant effect), where *P<0.05, **P<0.01.

Following our previous findings that CYP11A1-derived hydroxysecosteroids can inhibit proliferation of melanoma cell lines [49, 55], we selected 20(OH)D_3_ for further experimental testing on a SKMel-188 human melanoma line using established *in vitro* and *in vivo* assays of tumorigenesis and tumor progression. 20(OH)D_3_ inhibited colony formation in monolayer culture in a manner similar to 1,25(OH)_2_D_3_ (Figure 1A). The cytotoxicity of 20(OH)D_3_ in comparison to 1,25(OH)_2_D_3_ was tested using a novel spheroid toxicity assay, in which the contraction of spheroids formed through magnetic 3D bioprinting, was captured in real-time and utilized as a cytotoxic endpoint. This system was more representative of the 3D tissue environment than viability in 2D seen in an MTT assay. Once spheroids were formed, they began contracting immediately as a function of cell-cell interaction and cell-ECM (extracellular matrix) interaction, but the rate of contraction was slower in the presence of cytotoxic compounds [56]. 20(OH)D_3_ at a concentration of 10^-7^ M showed significant inhibition of spheroid contraction (Figure 1B) with 1,25(OH)_2_D_3_having no effect (not shown).

**Figure 2:**
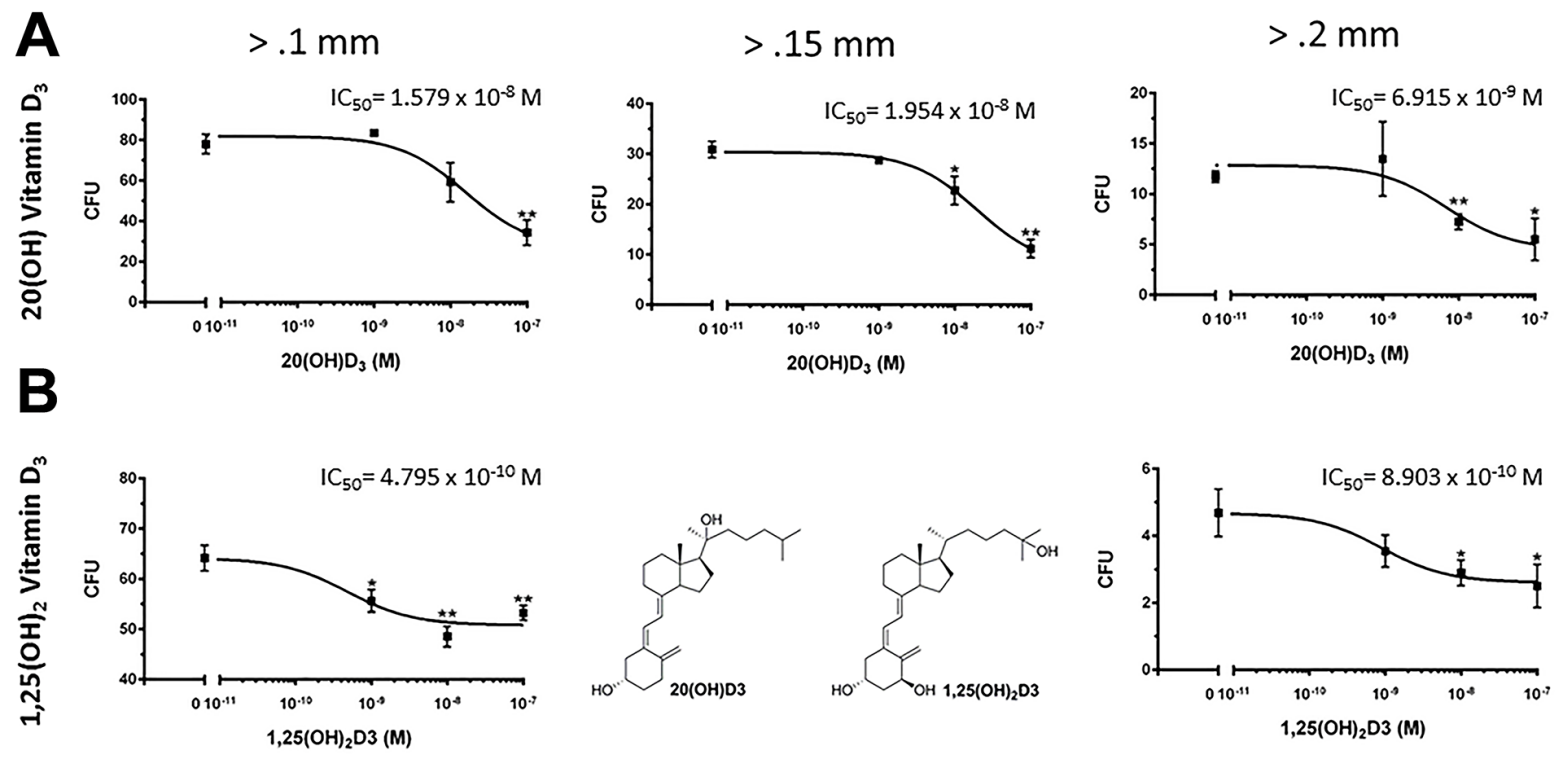
Effects of active forms on vitamin D on anchorage-independent growth of melanoma cells Both 20(OH)D_3_ and 1,25(OH)_2_D_3_ inhibit anchorage-independent growth in soft agar. Structures of 20(OH)D_3_ and 1,25(OH)_2_D_3_ are shown in panel B. SKMel-188 cells were seeded (500 cells/well) on a 0.8% agar layer and treated with 20(OH)D_3_ **A**. or 1,25(OH)_2_D_3_ **B**. (or ethanol vehicle as control) for 12 days. Colonies > 0.1 mm, 0.15 mm, and 0.2 mm were counted, where *P<0.05, **P<0.01.

**Figure 3:**
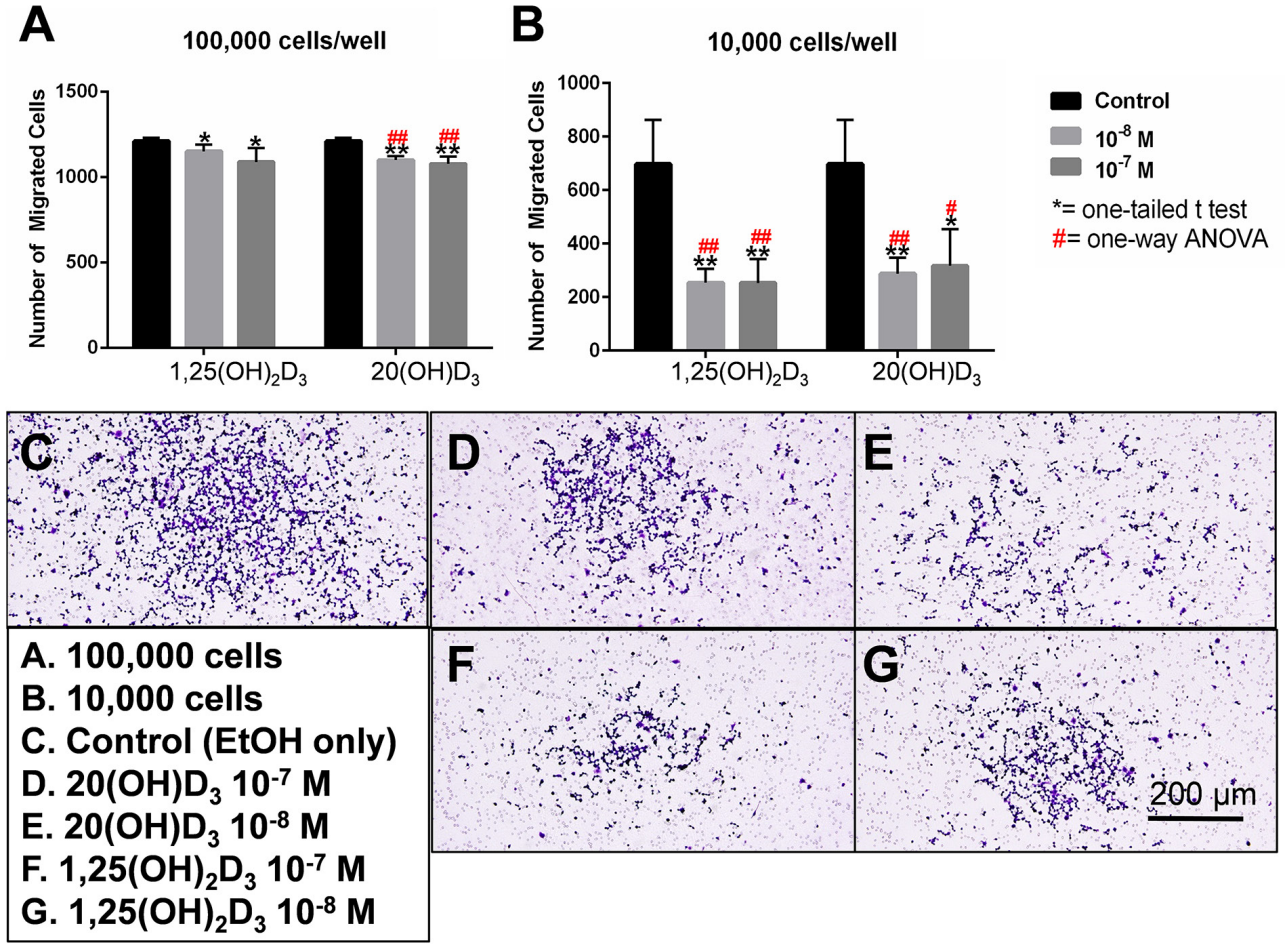
Effects of active forms on vitamin D on chemotactic capacity using a transwell migration assay SKMel-188 cells were placed on top of a transwell insert (polycarbonate membrane, pore size=8.0 μm) at a density of either 100,000 **A**. or 10,000 cells/well **B**. and the number of cells that migrated to the other side of the member in presence of a chemoattractant was quantified. The cells were incubated in the presence of graded concentrations of 20(OH)D_3_ **D, E**. or 1,25(OH)_2_D_3_ **F, G**. or ethanol as vehicle **C.** for 24 h. Top: 20(OH)D_3_ and 1,25(OH)_2_D_3_at 10^-7^ and 10^-8^ M inhibited chemotaxis in a dose-dependent manner at both cell densities (10,000 cells/well or 100,000 cells/well). The statistical significance was measured by a student *t*-test (*) or one way ANOVA (#); where (*, #) *P*<.05, (**, ##) *P*<.01. Bottom: A representative image from each treatment group (A-E) is shown.

Anchorage-independent growth, assessed by the formation of colonies with diameters >0.1mm, >0.15mm and >0.2 mm, was also inhibited by both 20(OH)D_3_ and 1,25(OH)_2_D_3_ in a dose-dependent manner (Figure 2). The IC_50_ values were lower in the groups treated with 1,25(OH)_2_D_3_ than 20(OH)D_3_ (0.48 × 10^-9^ M vs. 1.58 × 10^-8^ M, respectively for colonies > 0.1 mm and 0.89 × 10^-9^ M vs. 6.91 × 10^-9^ M, respectively for colonies > 0.2 mm. However, 20(OH)D_3_ showed higher efficacy in absolute percentage inhibition when comparing the inhibition at 10^-7^ M to that of 1,25(OH)_2_D_3_ (56.0% vs. 17.0%, respectively for colonies > 0.1 mm and 53.1% vs. 46.6%, respectively, for colonies > 0.2 mm). Furthermore, 20(OH)D_3_ significantly inhibited cell migration with an efficacy comparable to that of 1,25(OH)_2_D_3_ using the transwell migration assay at 100,000 and 10,000 cells/well (Figure 3).

### Antimelanoma activity of 20(OH)D_3_ in an *in vivo* model

**Figure 4:**
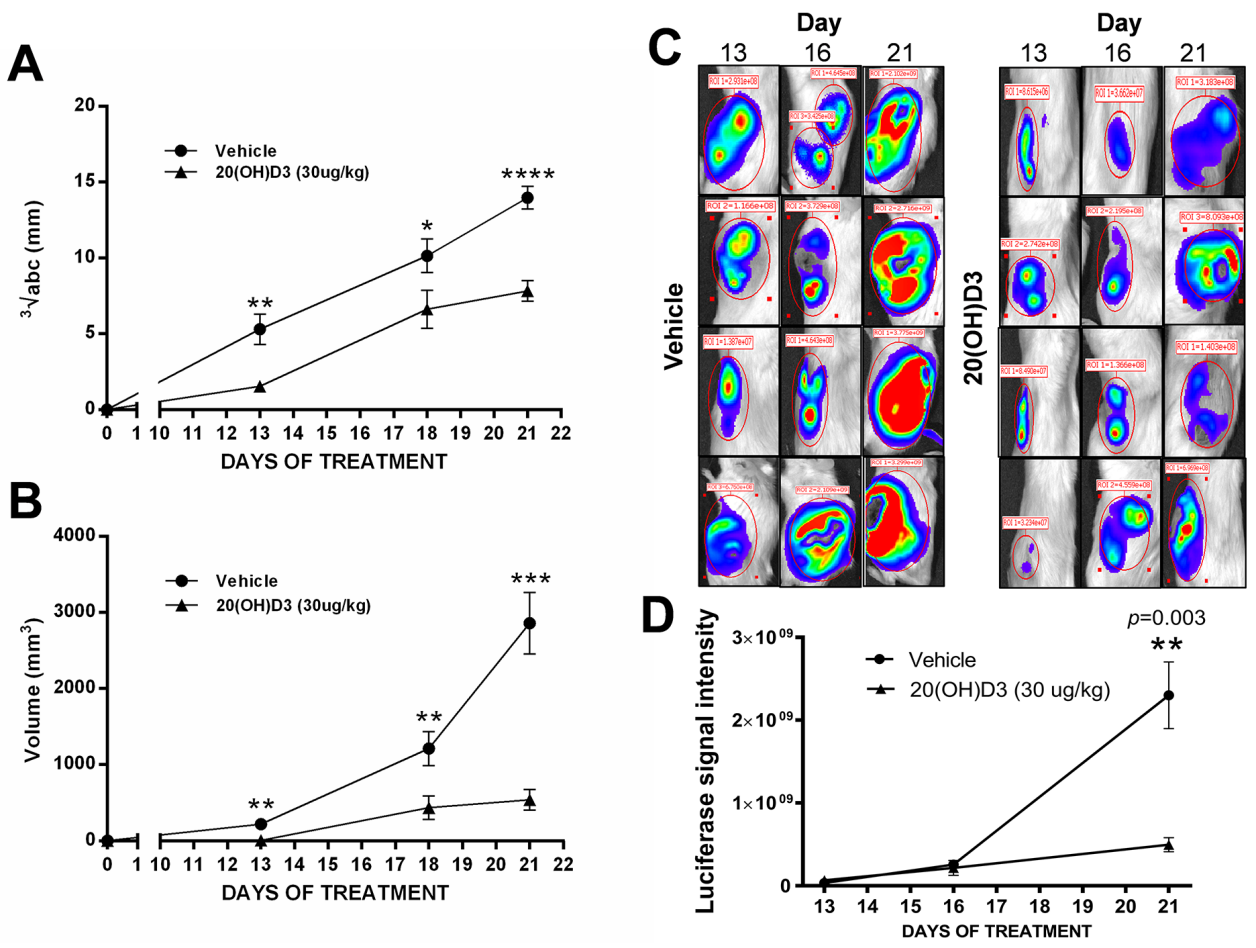
Treatment with 20(OH)D3 leads to an inhibition in growth of human melanoma in a mouse model The time dependent growth of implanted SKMel-188 melanomas is presented as geometric means of tumor dimensions **A**. and tumor volumes **B**. measured with caliper, where *P<0.05, **P<0.01, ***P<0.001, and ****P<0.0001; evaluated with a student's *t*-test to indicate the differences between control (vehicle) and 20(OH)D3 treatment. Representative examples (*n*=4 per each group) of luciferase intensity captured on day 13, 16, and 21 after tumor implantation are shown in **C**. with attendant calculation of the average signal intensity (*n*=10 from each group) in **D**.

Our *in vivo* study showed that the novel hydroxylated vitamin D_3_ derivative, 20(OH)D_3_, inhibits the growth of human melanoma in NSG mice. Of the twenty immunocompromised animals injected subcutaneously with SKMel-188 human melanoma cells, only mice that developed tumors were included for further study and analysis. Once the first palpable and measurable tumors (≥ 1 mm^3^) appeared on the 9^th^ day following implantation in 4 out of 20 mice (equal prevalence in control and treated group), the treatment with either 20(OH)D_3_ or vehicle began for all animals according to the randomization arm (see M&M). By the 12^th^ day following implantation (day 3 of treatment), 40% of animals had developed palpable tumors. All from both 20(OH)D_3_- and vehicle-treated mice (*n*=20) developed tumors by day 14 (5^th^ day of treatment). Sustained growth of tumors was subsequently observed in both groups, but tumor growth was impaired in 20(OH)D_3_-treated mice as compared to vehicle-treated mice. On day 6 of treatment, the accrued tumor volume was 262.35 mm^3^ in the 20(OH)D_3_-treated group versus 674.11 mm^3^ in the vehicle-treated group, representing a 61% decrease in tumor volume in the treated and control group, respectively. The inhibitory action of 20(OH)D_3_ on melanoma growth was statistically significant as measured by tumor volume or the geometric mean of the tumor dimensions on days 13, 18 and 21 post-implantation (Figure 4A, 4B).

The results of caliper evaluation of tumor growth in mice were further confirmed using bioluminescent imaging of tumor formation by melanoma cells expressing luciferase. The intensity of the luciferase signal indicates the number of viable melanoma cells and corresponds to tumor burden. Treatment with 20(OH)D_3_ led to a visible decrease in tumorigenesis when compared to control animals. Figure 4C, 4D shows representative images of 3 measurements (taken on 13, 16, and 21 day after tumor implantation) from 4 representative mice from each group, along with calculations of the signal intensity accumulated as a sum from all animals (*n*=10) in each group.

Although the survival rate was 90% in both groups on the last day of experiment, the total body score representing the health status of the animals was higher in the 20(OH)D_3_-treated group compared to controls (2.8 vs 2.55, respectively). Body condition scoring is a practical, rapid, noninvasive method for assessing health status in animals. Changes in body weight, behavior and physical appearances, commonly suggested as standard indicators of health or illness, were recorded during observation and palpation performed by a veterinarian. A mouse of BC3 status was in optimal condition and any increase/decrease in BC value indicated deterioration of health. No significant inhibitory effect on melanoma growth was observed in animals treated with a lower (3 μg/kg) dose of 20(OH)D_3_ (data not shown).

**Figure 5:**
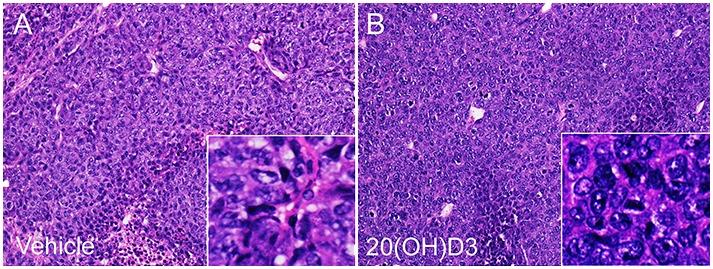
Histology of the implanted tumors Panels A and B show representative H&E stained sections of tumors from animals treated with either vehicle **A**. or 20(OH)D3 **B**. Microscopic magnification of main panels: x 12.5; insert: x 45.

The morphology of implanted melanomas is shown in Figure 5. H&E stained sections showed that tumors treated either with vehicle or 20(OH)D_3_ were composed of pleomorphic amelanotic melanoma cells with epithelioid morphology with pleomorphic hyperchromatic nuclei, frequent mitoses and areas of necrosis (Figure 5).

## DISCUSSION

20(OH)D_3_ is the major metabolite of a newly discovered CYP11A1-mediated hydroxylation pathway of vitamin D_3_ activation [43, 45, 57, 58], which operates *in vivo* in humans [44]. 20(OH)D_3_ is noncalcemic and non-toxic at high pharmacological doses (30-60 μg/kg in rodents) [53, 54], which are approximately 300-600 times higher than doses of 1,25(OH)_2_D_3_ that produce hypercalcemia [38, 50]. Previously, we reported anticancer and antimelanoma activities of 20(OH)D_3_ *in vitro* [48, 49, 51–53, 55]. In accordance with our prior findings, this current study showed that 20(OH)D_3_ inhibits colony formation by SKMel-188 cells in soft agar assays, indicating that it reduces anchorage-independent growth of melanoma cells. Additionally, we showed that 20(OH)D_3_ inhibits cell migration, and cell-cell and cell-extracellular matrix interactions in both a transwell migration assay that assesses the chemotactic capability of cells, and a spheroid toxicity assay that assesses the migratory capability, cell-cell interactions and cell-ECM interactions [56, 59]. Lastly, the effects of 20(OH)D_3_ were observed *in vivo* since it decreased melanoma tumor in immunocompromised mice, without visible signs of toxicity.

The chemotactic capability and migratory capability are both important metastatic parameters that become unregulated and facilitate cancer cell migration [60]. Furthermore, the loss of intracellular adhesion and abnormalities in cell-matrix interaction are also critical in the progression stage of carcinogenesis [61]. Developing a therapy targeting tumor progression of melanoma is essential since metastatic malignant melanoma has an especially poor prognosis, even with the advent of new molecularly targeted therapeutic modalities. 20(OH)D_3_ demonstrated antimelanoma activity comparable to 1,25(OH)_2_D_3_ in the transwell migration assay, and demonstrated superior antimelanoma activity in the spheroid toxicity assay.

The partial success of the recently introduced treatment, aimed at PD-1/PD-L1 blockade limited by the lack of a sufficient number of activated lymphocytes, encouraged scientists to search for more reliable and efficient models and treatments for melanoma. The gold standard for melanoma study is the patient-derived orthotopic xenograft model which enables the tracking of tumor complexity that mimics the behavior in “real” *in vivo* environment [62]. The immunocompromised (nude) mouse is a well-established animal model to study human melanoma [63–66]. Tumor growth is a complex process, ultimately dependent on the environment of the proliferating tumor cells in the host tissues. The current view of tumor growth kinetics is based on the general assumption that tumor cells grow exponentially. However, a discrepancy is evident between the exponential tumor growth theory and *in vivo* experimental data, since tumor doubling times have been found to greatly exceed cell cycle times [67]. This may explain why tumors occurred in our study at a different time (9 to 14 days) after inoculation. Usually it takes 5 to 10 days to reach a volume of 1 mm^3^ with an injection of approximately 10^5^ – 10^6^ cells [64, 68]. However, the murine melanoma model is unpredictable with regard to the specific timeline of tumor development. This unpredictability is particularly relevant for defining a strict criterion for tumor diagnosis (size or volume) and for setting a starting day for drug treatment. Thus, we employed two independent methods of tumor measurement (caliper and bioluminescence) and two methods of presenting values (tumor volume and a geometric mean of the tumor dimensions) in our experiments to provide a reliable track of tumor growth, as recommended for assuring the validity of an *in vivo* study [69, 70]. All of these methods showed attenuation of tumor growth by 20(OH)D_3_ at the dose of 30 μg/kg without any sign of toxicity. Another robust “tool” to track progressiveness of metastatic melanomas is the GFP-transduced melanoma model [71]. Naturally fluorescent proteins have revolutionized biology in the way that single metastatic cell can be observed and tracked even at the subcellular level [72, 73]. Moreover, the combination of GFP-expressing melanomas and transgenic mice displaying traced blood vessels along with the application of the noninvasive imaging [74] may be extremely advantageous method for future studies.

The total body score that assessed animal health, was higher in the treatment compared to the control group (2.8 vs. 2.55). The better overall health condition observed after 20(OH)D_3_ treatment demonstrated as closer to optimal 3 BC score than for untreated animals and may be a useful paradigm describing the tolerability of novel compounds under investigation. Although the direct extrapolation of rodent models to human well-being is questioned by many investigators, it is worthy of consideration in designing novel preclinical studies.

1,25(OH)_2_D_3_, acting through the VDR, causes hypercalcemia leading to failure of multiple organs at doses as low as 0.1 μg/kg [38]. Also, 25(OH)D_3_ induces hypercalcemia in animals lacking the 1α-hydroxylase (CYP27B1 −/−knock-out) at relatively low doses [38]. 20(OH)D_3_ at a dose of 30 μg/kg did not cause side effects in our experiment whereas doses of 1,25(OH)_2_D_3_ and 25(OH)D_3_ 300 and 30 times lower than this, respectively, caused hypercalcemic (0.1-3 μg/kg), as we previously reported [50]. The lack of side effects of 20(OH)D_3_ is consistent with our previous testing on mice which showed that 20(OH)D_3_ is non-calcemic at doses as high as 30-60 μg/kg [53, 54]. These studies identify 20(OH)D_3_ as an excellent therapeutic or adjuvant agent that is non-toxic and non-calcemic at least at 30 μg/kg.

It is accepted that the phenotypic activity of 1,25(OH)_2_D_3_ is mediated by its interaction with the VDR [31, 32, 75, 76] which can be defined as the canonical signal transduction pathway. Similarly, non-calcemic 20(OH)D_3_ can also activate the VDR on melanoma cells [48, 58, 77] causing downstream anti-proliferative and antitumorigenic effects. Previously, we have demonstrated that 20(OH)D_3_ and its metabolites can inhibit NF-κB signaling through an interaction with VDR in keratinocytes [78, 79] and melanoma cells [47]. Thus, 20(OH)D3 can inhibit melanoma growth through inhibition of NF-κB in tumor cells and/or by reduction of proinflammatory activity in the stroma. Recently we discoverd that 20(OH)D_3_ also act on retinoic acid orphan receptors (ROR) α and γ as a inverse agonist [80], and that these receptors are expressed in human melanomas [80, 81], suggesting their involvement in the inhibition of melanoma growth by 20(OH)D_3_ through a non-canonical pathway. The relative involvement of the VDR or RORα and γ in the inhibition of melanoma growth by 20(OH)D_3_ represents a challenging goal for future investigation. In this context, it must be noted that clinic-pathological studies have shown a correlation between changes in the expression of VDR or RORs with melanoma progression and also with overall and disease free survival time of the patients [9, 81, 82].

In conclusion, we provided *in vitro* and *in vivo* evidence that the CYP11A1-derived 20(OH)D_3_ is an excellent candidate for further testing as a primary or adjuvant therapeutic agent against human melanoma.

## MATERIALS AND METHODS

### Vitamin D_3_ hydroxyderivatives

1,25(OH)_2_D_3_ was obtained from Fluka Chemicals (Sigma-Aldrich, St. Louis, MO). 20(OH)D_3_ was produced by the enzymatic hydroxylation of vitamin D_3_ by CYP11A1 as described previously [40, 57]. The extracted product was purified by preparative thin-layer chromatography followed by reverse phase HPLC. The structure was confirmed by NMR as detailed in [40, 57]. The D_3_ hydroxyderivatives were dried and stored at –80°C until use.

### Culture of the SKMel-188 melanoma cell line

A human melanoma line (SKMel-188) was obtained from Dr. Chakraborty from Yale University. Since then, it had been characterized and maintained in our laboratory [49, 83, 84]. This cell line was used for assays of plating efficiency, colony formation in soft agar, spheroid toxicity, and transwell migration. The line was grown at 37°C in a humidified atmosphere with 5% CO_2_. Ham's F10 medium supplemented with glucose, L-glutamine, pyridoxine hydrochloride (Cellgrow, Manassas, VA), 5% charcoal-treated fetal bovine serum (CS-FBS) (Sigma, St. Louis, MO) and 1% penicillin/ streptomycin/amphotericin antibiotic solution (Sigma, St. Louis, MO) was used to culture the cells. In addition, human SKMEL-188 cells were transduced with a lentiviral luciferase (LUC) construct for live animal bioimaging analysis for tumor formation as described previously [85].

### Plating efficiency of SKMel-188 cells

SKMel-188 cells were plated on a 6-well plate at a density of 14 cells/cm^2^ in Ham's F10 medium supplemented with 5% CS-FBS. 1,25(OH)_2_D_3_ or 20(OH)D_3_ (or ethanol vehicle as a control) was added to each well every 72 h to a final concentration of 10^-7^ M. Each condition was tested at least in triplicate. After 14 days, colonies were fixed in 4% paraformaldehyde and stained with 2.3% crystal violet (Sigma-Aldrich, St. Louis, MO). Colonies were imaged using the Cytation 5 Cell Imaging Multi-Mode Reader and quantified using the Gen5 software (Biotek, Winooski, VT). The number of colony-forming units (CFU) was calculated for each condition using the following formula: CFU = 100[(number of colonies)/(number of cells plated)].

### Colony formation in soft agar

The melanoma cells were trypsinized, re-suspended in medium containing 0.4% agarose (American Bioanalytical, Natick, MA) and 5% CS-FBS and seeded at 500 cells/well in a 0.8% agar layer on 24-well plates. 20(OH)D_3_ or 1,25(OH)_2_D_3_ (or ethanol vehicle as a control)was added to each well to final concentrations of 10^-7^ M, 10^-8^ M, or 10^-9^ M using 100 μL of media supplemented with 5% CS-FBS. Each condition was tested at least in duplicate. Every 72 h over 12 days, fresh 20(OH)D_3_or 1,25(OH)_2_D_3_ (or ethanol control) in 100 μL medium, was added. Subsequently, resulting colonies were stained with 0.5 mg/ml of 5-(dimethylthiazol-2-yl)-2,5-diphenyltetrazolium bromide (MTT) reagent (Promega, Madison, WI), imaged using the Cytation 5 Cell Imaging Multi-Mode Reader in 3 different z-planes and scored using Gen5 software. The number of CFU was calculated for each condition.

### Spheroid toxicity

The procedure followed was based on a more detailed protocol (1). Briefly, melanoma cells at 70-80% confluence were incubated with magnetic nanoparticles (NanoShuttle) containing gold, iron oxide, and poly-L-lysine (Nano3D Biosciences, Houston, TX) at a concentration of 50 pg/cell, overnight. This process allowed the cells to become magnetized. Subsequently, the cells were trypsinized, resuspended in medium at a concentration of 1.6 × 10^6^ cells/mL (3.2 × 10^6^ cells in 2 mL) and dispensed into an ultra-low attachment 6-well plate (Corning, Tewksbury, MA). A magnetic drive of 6 neodymium magnets (Nano3D Biosciences) was placed on top of the 6-well plate to allow the cells to levitate and to induce the formation of extracellular matrix (ECM). After 24 h, the levitated cultures were broken up using rigorous pipette action. Melanoma cells (50,000 cells/well) were then dispensed into an ultra-low attachment 96-well plate (Corning). A magnetic drive of 96 neodymium magnets was placed below the 96-well plate to print the cells into spheroids over 1 h. 1,25(OH)_2_D_3_ or 20(OH)D_3_ (or ethanol vehicle as a control) was added to each well at final concentrations of 10^-7^ or 10^-8^ M. Each condition was tested at least in triplicate. The magnet was subsequently removed to allow the spheroids to contract for 48 h. Images were automatically taken every hour on a mobile device, iPod touch 5th generation, 16 GB (Apple Computer, Cupertino, CA) using an application (Experimental Assistant, Nano3D Biosciences). The images were then analyzed to calculate the area of each spheroid using a custom analysis code in Python provided by Nano3D Biosciences.

### Transwell migration assay

The HTS Transwell-24 System (Corning) was used to carry out the assay as per the manufacturer's instructions. Serum-free Ham's F10 medium was added to each well and to each transwell insert; the plate was incubated for 1 h to provide an initial equilibration period. The melanoma cells were harvested, resuspended in Ham's F10 medium supplemented with 1% CS-FBS and 100 μL of the cell suspension was dispensed in an insert of the transwell plate (polycarbonate membrane, pore size=8.0 μm). Two separate conditions with varying cell densities, 10,000 cells/well and 100,000 cells/well, were tested. 20(OH)D_3_ or 1,25(OH)_2_D_3_ (or ethanol vehicle or medium as controls) was added to each insert to final concentrations of 10^-7^ M or 10^-8^ M. Six hundred μL of 10% CS-FBS were then added to the lower chamber to serve as a chemoattractant. After 24 h, a cotton-tipped applicator was used on top of the membrane to remove excess cells and media. The inserts were then washed in chilled PBS for 2 min, fixed with ice-cold methanol for 10 min and stained using 2.3% crystal violet. The inserts were then washed in distilled water twice for 5 min, air-dried and mounted using Permount (Fisher Scientific, Pittsburgh, PA). Each experiment condition was tested in duplicate. The slides were then imaged using the Cytation 5 Cell Imaging Multi-Mode Reader and the migrated cells were quantified using Gen5 software in 3 separate 10X fields in color bright field (6 separate fields/condition).

### Animal experiments

All animal experiments were performed in accordance with a study protocol approved by the Institutional Animal Care and Use Committee of the University of Tennessee Health Science Center (UTHSC). Seven-week-old female NOD.Cg-*Prkdc^scid^ Il2rg^tm1Wjl^*/SzJ (NSG) mice (Jackson Laboratory) were placed on a vitamin D-deficient diet (TD.89123, Harlan Laboratories, Madison, WI). After two weeks, animals were randomly divided into 2 groups (*n*=10) and injected subcutaneously into the flanks with 1×10^6^ luciferase-expressing SKMel-188 cells. Tumor initiation and progression were monitored twice a week after D-luciferin injection using the Xenogen IVIS (Perkin Elmer, Waltham, MA) bioimaging system as well as measured with a caliper. Tumor size, expressed as a total volume, was calculated using the formula: a x b x c, and was provided in mm^3^ [67] or as (geometric mean) a×b×c3; where a, b and c represented length, width and depth, respectively [70, 86]. Once implanted tumors became palpable and reached 1 mm^3^ in size, the treatment was started with intraperitoneal injection of a matched volume of either 30 μg/day of 20(OH)D_3_ diluted in vehicle (25 % propylene glycol in distilled water) or vehicle, per animal. Ten doses in total, 5 days per week, were applied for 2 weeks. The body score (BS) scale, representing the overall health condition of animal, was performed bi-weekly [87]. Three days following the last treatment (day 21^th^), animals were sacrificed with CO_2_ followed by cervical dislocation and their organs were collected for macroscopic and microscopic evaluation.

### Statistical analysis

All statistical analyses were performed using GraphPad Prism Version 6.0 (GraphPad Software, San Diego, CA). For the assays of colony formation in soft agar and plating efficiency, the digital data obtained from the Gen5 software were analyzed using a *t*-test, and an IC_50_ for each condition was calculated. For the transwell migration assay, the digital data obtained from the Gen5 software was analyzed using a *t*-test and a one-way ANOVA test with a *post hoc* Tukey's test. For the spheroid toxicity assay, the area of each spheroid, directly measured using the images acquired from a mobile device, was plotted and analyzed using a one-tailed *t*-test. Differences were considered statistically significant when *P* < 0.05. The data are presented as mean ± standard error. The *t*-test was employed for comparing the measurement of *in vivo* tumor growth between the vehicle (control) and 20(OH)D_3_ treatment, and considered statistically significant at *P* < 0.05 (*), *P* < 0.01 (**), *P* < 0.001 (***), and *P* < 0.0001 (****).
